# Changing Patterns and Driving Factors of Plankton Coupling Relationships in Lakes around the Yangtze River, China

**DOI:** 10.3390/microorganisms12081698

**Published:** 2024-08-17

**Authors:** Chenhao Dong, Xinchao Guo, Haiyan Liu, Zhaosheng Chu, Tianhao Wu

**Affiliations:** 1 National Engineering Laboratory for Lake Pollution Control and Ecological Restoration, Chinese Research Academy of Environmental Sciences, Beijing 100012, China; dchgr97@126.com (C.D.);; 2 State Environmental Protection Key Laboratory for Lake Pollution Control, Chinese Research Academy of Environmental Sciences, Beijing 100012, China; 3 School of Environmental and Municipal Engineering, Xi’an University of Architecture and Technology, Xi’an 710055, China

**Keywords:** Yangtze River, zooplankton, phytoplankton, predation, cyanobacteria

## Abstract

In recent decades, cyanobacterial blooms have intensified in many lakes in China. Algal blooms are closely linked to the predation pressure on phytoplankton, but the changes in the relationship between phytoplankton and their primary predators, zooplankton, remain unclear. To investigate the changing patterns and driving factors of the relationship between plankton, the historical data of plankton from 14 typical freshwater lakes around the Yangtze River were collected from multiple databases. By comparing the structure of plankton communities in typical lakes between the 1990s and the 2010s, it was found that the phytoplankton density was elevated in 79% of all the lakes; on average, it had increased to 3156 times higher than it had been. In contrast, the zooplankton density was elevated in only 57% of these lakes, and this value was only two times higher than it had been. In 11 out of the 14 lakes, the zooplankton density growth rate was lower than that of the phytoplankton. The percentage of cyanobacteria in these lakes increased from 53% to 62%, and the changes in cyanobacteria were significantly negatively correlated with the changes in zooplankton. Eutrophication caused this significant increase in phytoplankton, especially cyanobacteria. Cyanobacterialization, changes in fish community structures, biological invasion, and river–lake relationships impede zooplankton survival. This combination of factors hinders plankton coupling in many lakes. This study attempts to provide new insights for lake ecological management.

## 1. Introduction

Phytoplankton are one of the important primary producers in lake ecosystems. A healthy phytoplankton community can provide the necessary materials and energy for lake ecosystems, but the proliferation or aggregation of phytoplankton may lead to algal blooms [1,2]. One of the most common algal blooms is the cyanobacterial bloom. Algal blooms are phenomena caused by the rapid proliferation or aggregation of phytoplankton, resulting in the discoloration of water bodies. Many freshwater lakes in China have experienced algal blooms in recent decades, leading to severe ecosystem degradation [3,4].

Zooplankton are an important component of lake ecosystems. Zooplankton are the key predators of phytoplankton, assimilating materials and obtaining energy through predation. Fish prey upon and consume zooplankton, positioning them as the middle link in the lake food web [5]. The fish community structure strongly influences zooplankton; zooplanktivorous fish feed directly on zooplankton, while omnivorous and phytophagous fish affect zooplankton indirectly by influencing phytoplankton, aquatic plants, and other biological components [6]. Cyanobacteria have an inhibitory effect on zooplankton. In lakes with excessive cyanobacteria, cyanotoxins and hypoxia threaten the survival of zooplankton, and cyanobacteria can resist predation by crustacean zooplankton by aggregating into clusters [6,7].

The predation relationship between zooplankton and phytoplankton forms the fundamental part of the lake food web. In healthy lake ecosystems, there is a dynamic balance between zooplankton and phytoplankton [8,9]. Other studies have shown that algal blooms are related to the balance between zooplankton and phytoplankton [10]. Many researchers have studied lakes in the United States, Canada, Belgium, and other countries, and most have found that the phytoplankton community structure strongly influences plankton coupling [11,12,13]. The ratio of zooplankton to phytoplankton density or biomass (Z/P) is an important indicator for evaluating the health of lake ecosystems [14,15]. The Z/P ratio is also known as the resource utilization efficiency (RUE), and it shows whether the phytoplankton biomass has been efficiently transferred to the next trophic level [3]. Recent research has shown that a decrease in the Z/P ratio is the key indicator of algal blooms in lakes [16].

The Yangtze River Basin is the most developed region in China, but many surrounding lake ecosystems have been harmed and algal blooms are being exacerbated. The percentage of cyanobacteria in many lakes has increased dramatically, and cyanobacterization has intensified. Many researchers have studied this phenomenon, but most studies focused only on the bottom-up effect; few studies concentrated on why zooplankton fail to control phytoplankton. Both the bottom–up and top–down effects are important in controlling algal blooms. It is well known that algal bloom occurrence is closely related to the predatory relationship among plankton, but, in fact, the relationship between phytoplankton and zooplankton in these lakes is still unclear. To determine what causes the changes in the relationship between phytoplankton and zooplankton in lakes around the Yangtze River, we collected over 30 years of data from 14 typical lakes in this region. We analyzed and attempted to reveal the characteristics and factors driving the changes in the predation relationship between zooplankton and phytoplankton.

## 2. Methods

### 2.1. Study Area

The Yangtze River is the largest river in China, with a basin area of 1.8 × 10^6^ km^2^, accounting for about 20% of the country’s total area. There are 145 lakes over 10 km^2^ in the Yangtze River Basin, with the lake basin having a population of about 270 million and a GDP that accounts for 21.9% that of China.

### 2.2. Data Sources

**Figure 1:**
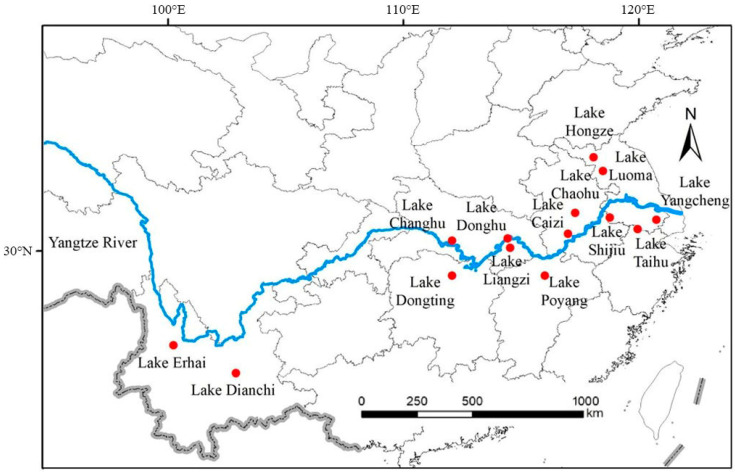
The location of the 14 studied lakes around the Yangtze River.

We focused on 14 well-studied lakes that are important to humans (Figure 1). The upper reaches of the Yangtze River include typical plateau lakes such as Lake Dianchi and Lake Erhai. The middle reaches include Lake Poyang, Lake Dongting, Lake Changhu, Lake Liangzi, and Lake Donghu. The lower reaches include Lake Chaohu, Lake Taihu, Lake Caizi, Lake Shijiu, Lake Yangcheng, Lake Hongze, and Lake Luoma.

This study collected data during 1990–2023 from multiple databases, including the China National Knowledge Infrastructure (CNKI), Web of Science (WOS), China National Ecosystem Observation and Research Network (CNERN), and lake monitoring stations (Table 1). The plankton density, biomass, community structure, water quality, and socio-economic data were extracted for analysis. The data from the 1990s and 2010s were comparatively analyzed. CNERN provided data from the 2007–2019 annual survey of the plankton community structure in Lake Taihu and Lake Donghu. Only data from annual lake-wide surveys were used to ensure representativeness and reliability. All studies used microscopes and hemocytometers to count the phytoplankton, and the phytoplankton volume and density were combined to calculate the biomass. The major cities upstream and near the lakes featuring human activities having a huge impact were identified, and the GDP was obtained through city yearbooks and government work reports. The GDP of these cities was calculated and correlated with the plankton.

### 2.3. Analysis Methods

The Trophic Level Index (*TLI*) was calculated based on 5 parameters: total phosphorus (*TP*), total nitrogen (*TN*), chlorophyll a (*chla*), transparency (*SD*), and the permanganate index (*COD_Mn_*) [40]. The calculation method was as follows, and $r_{ij}^{2}$ were showed in Table 2.

$$\left\{\begin{array}{c} TLI=\sum_{j=1}^{m}W_{j}\times TLI(j)\\ W_{j}=\frac{r_{ij}^{2}}{\sum_{j=1}^{m}r_{ij}^{2}} \end{array}\right.$$

$$\left\{\begin{array}{c} TLI\left(chla\right)=10\times (2.5+1.086\ln chla)\\ TLI\left(TP\right)=10\times (9.436+1.624\ln TP)\\ TLI\left(TN\right)=10\times (5.453+1.694\ln TN)\\ TLI\left(SD\right)=10\times (5.118-1.94\ln SD)\\ TLI\left({COD}_{Mn}\right)=10\times (0.109+2.661\ln{COD}_{Mn}) \end{array}\right.$$

The Z/P ratio indicates the relationship between zooplankton and phytoplankton. The Z/P ratio of a lake in the 1990s was used as the reference for comparison with the same lake in the 2010s to determine how it had changed. Since the plankton community structures of different lakes vary greatly, we did not compare the Z/P ratio between lakes, but rather the differences in the Z/P ratio at different periods for the same lake.

We used ArcGIS 10.4 to map the distribution of the lakes. Bar charts and line graphs were drawn with Origin 2018. Correlation analysis was conducted with SPSS 24 to analyze the relationships between the phytoplankton densities, GDP, the plankton, the Z/P ratio, and the water quality. The data distribution was checked before correlation analysis. Pearson correlation was used for normal distributions, and Spearman correlation was used for non-normal distributions. Redundancy analysis was performed using the R 4.0.3 and vegan packages. The environmental factors were filtered according to the variance inflation factor (VIF); those with a VIF greater than 10 were removed one by one.

## 3. Results

### 3.1. Trophic Level of Lakes in the Lake Basins

**Figure 2:**
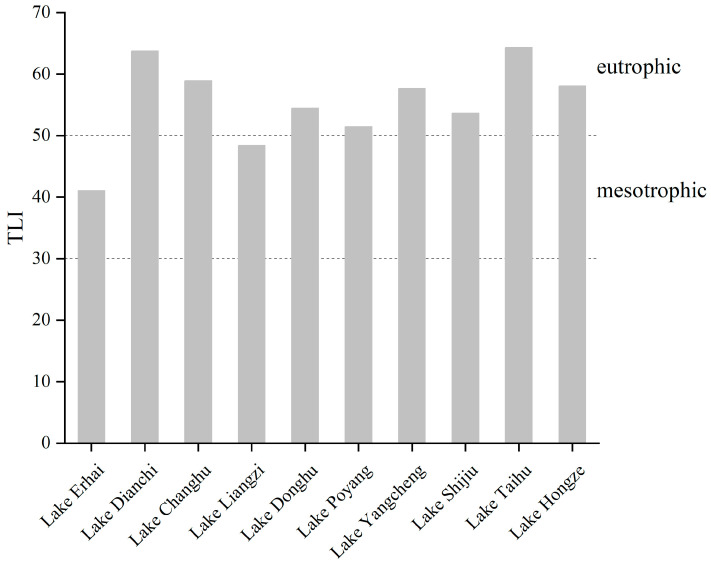
TLI of the studied lakes.

In the 2010s, most lakes around the Yangtze River were eutrophic; only Lake Erhai and Lake Liangzi were mesotrophic (Figure 2). Lake Dianchi and Lake Taihu had a TLI of 64 in the 2010s, indicating moderate eutrophication. The nutrient and chlorophyll a concentrations were high and transparency was low in both lakes. Lake Erhai and Lake Liangzi had TLIs of 41 and 48, respectively, indicating mesotrophy. The nutrient concentrations were low and transparency was high in both lakes, despite the chlorophyll a concentrations not being low.

### 3.2. Changes in Plankton Coupling Relationships in Lakes around the Yangtze River

#### 3.2.1. Changes in the Phytoplankton Community Structure

**Figure 3:**
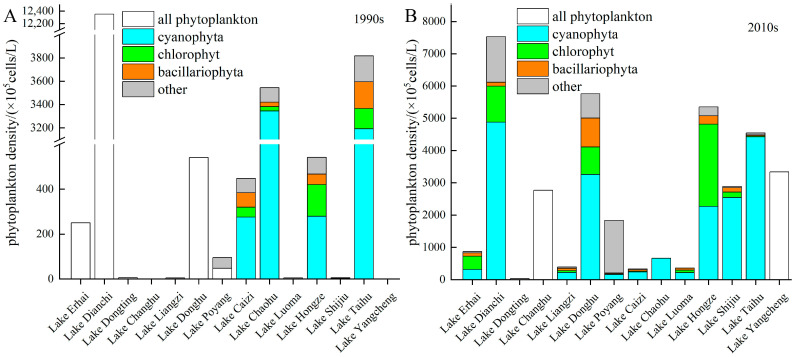
Phytoplankton density and community structure in the (**A**) 1990s and (**B**) 2010s.

From the 1990s to the 2010s, the phytoplankton density increased significantly in 11 out of the 14 lakes, with only Lake Chaohu, Lake Caizi, and Lake Dianchi showing a decrease. The average phytoplankton density across the 14 lakes increased by 3156 times (Figure 3). The largest increase occurred in Lake Changhu, where the phytoplankton density rose from 7.0 × 10^2^ cells/L in the 1990s to 2.7 × 10^7^ cells/L in the 2010s. The density of phytoplankton in Lake Yangcheng and Lake Shijiu increased by 3882 and 481 times, respectively. That in Lake Liangzi, Lake Poyang, Lake Luoma, Lake Donghu, and Lake Dongting was 45 times higher on average. The density in Lake Hongze, Lake Taihu, and Lake Erhai was 10 times higher on average. Only Lake Caizi, Lake Chaohu, and Lake Dianchi showed a slight decrease in density.

In terms of phytoplankton community composition, cyanobacteria comprised 53% of the phytoplankton in the 1990s, reaching 62% in the 2010s. In the 2010s, the percentage of cyanobacteria in Lake Luoma, Lake Liangzi, and Lake Dianchi exceeded 50%, and that in Lake Taihu, Lake Shijiu and Lake Caizi even exceeded 70%.

#### 3.2.2. Changes in the Zooplankton Community Structure

**Figure 4:**
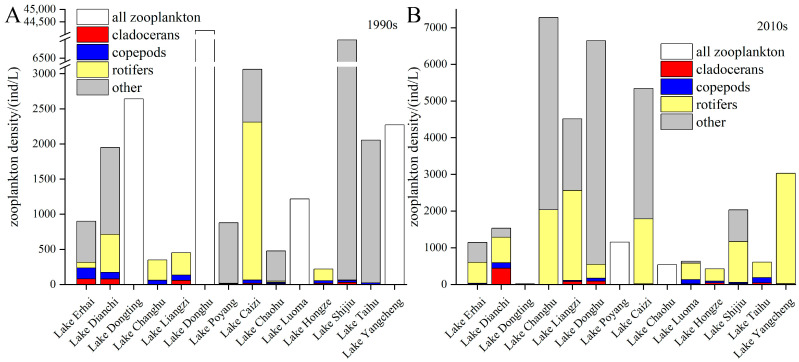
Zooplankton density and community structure in the (**A**) 1990s and (**B**) 2010s.

The average zooplankton density in the lakes from the 1990s to the 2010s doubled, which was much lower than that of the phytoplankton. The zooplankton densities increased in eight lakes and decreased in six lakes from the 1990s to the 2010s (Figure 4). The largest increases appeared in Lake Liangzi and Lake Changhu, from 453 and 349 ind/L to 4512 and 7280 ind/L, respectively. The density in Lake Hongze, Lake Yangcheng, Lake Chaohu, Lake Caizi, Lake Poyang, and Lake Erhai increased less than one-fold. Among the lakes with decreasing zooplankton densities during the 1990s–2010s, the zooplankton density of Lake Dongting decreased from 2641 to 20 ind/L, which was a decrease of 99%. The zooplankton density of Lake Donghu decreased from 44160 ind/L to 6645 ind/L, which was a decrease of 85%. The zooplankton densities of Lake Taihu and Lake Shijiu decreased by 70%, while those of Lake Luoma and Lake Dianchi decreased by 48% and 27%, respectively.

In terms of the zooplankton community structure, the average proportion of rotifers increased from 38% to 54% from the 1990s to the 2010s, while that of crustacean zooplankton remained almost unchanged. The average proportion of cladocerans among the zooplankton in the 1990s was 4.6%. Among them, Lake Liangzi had the highest proportion of cladocerans at 13.0%, and that of both Lake Erhai and Lake Hongze was about 8%. By the 2010s, the proportion of cladocerans had increased to 6.1%, with 10.3% in Hongze Lake and 7.1% in Lake Shijiu. The proportion of copepods was 7.3% in 1990s, with the average in Lake Hongze, Lake Erhai, Lake Liangzi, and Lake Changhu being 17.0%. The average proportion of copepods in the 2010s was 6.9%, and this was more than 10% in Lake Hongze, Lake Luoma, and Lake Taihu.

#### 3.2.3. Changes in the Z/P Ratio

**Figure 5:**
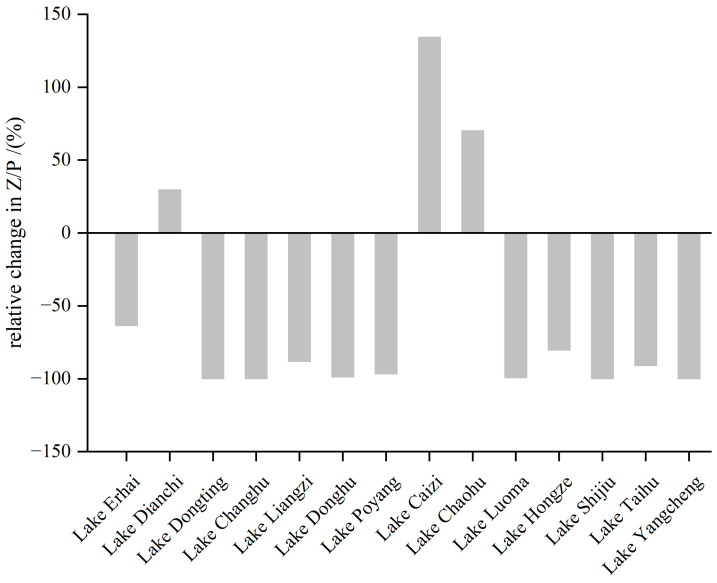
Relative changes in the zooplankton/phytoplankton densities.

During the 1990s–2010s, 79% of the lakes around the Yangtze River showed a decreasing trend in the Z/P ratio. The average decrease in the Z/P ratio in these 79% lakes was as high as 92% (Figure 5). The Z/P ratio decreased by more than 90% in Lake Dongting, Lake Changhu, Lake Donghu, Lake Poyang, Lake Yangcheng, Lake Shijiu, Lake Taihu, and Lake Luoma. The increase in zooplankton in these lakes did not effectively impede the rapid growth of phytoplankton. The average decrease in Lake Liangzi, Lake Erhai, and Lake Hongze also reached 73%. The zooplankton did not effectively prey on the phytoplankton in these lakes. Only Lake Chaohu, Lake Caizi, and Lake Dianchi showed 70%, 134%, and 30% increases in the Z/P ratio, respectively.

During the 1990s–2010s, the changes in the Z/P ratio varied among the lakes, but they were mostly caused by changes in the phytoplankton. Among them, the phytoplankton density of Lake Luoma, Lake Shijiu, Lake Yangcheng, Lake Poyang, Lake Donghu, Lake Liangzi, Lake Changhu, and Lake Dongting increased by more than 10 times. During the same period, the zooplankton density of eight lakes, except for Lake Liangzi, only increased by less than 1-fold or even decreased; the decoupling of phytoplankton and zooplankton growth in these lakes led to the significant decrease in the Z/P ratio. The phytoplankton densities in Lake Hongze, Lake Taihu, and Lake Erhai were elevated 9-, 1-, and 2-fold, respectively. However, during the same period, the zooplankton densities were elevated by 95%, decreased by 81%, and elevated by 27%, respectively. The zooplankton density in these lakes increased more slowly than that of phytoplankton, resulting in a decrease in the Z/P ratio. The phytoplankton density in Lake Chaohu, Lake Caizi, and Lake Dianchi decreased by 33%, 25%, and 39% respectively. During the same period, the zooplankton density increased by 13%, 75%, and 21%, respectively. The effective control of phytoplankton and zooplankton density caused the Z/P ratio of these lakes to increase.

### 3.3. Long-Term Change in the Z/P Ratio in Lake Taihu and Lake Donghu

#### 3.3.1. Lake Taihu

The phytoplankton biomass in Lake Taihu increased dramatically from 2007 to 2019, while the zooplankton biomass decreased during the same period. The phytoplankton biomass was 0.31 mg/L in 2007, and then it increased in 2009. The phytoplankton biomass was as high as 0.66 mg/L in 2014, and it remained stable in the years that followed. During 2007–2019, the phytoplankton biomass was elevated by 113%. The zooplankton biomass was 13.22 mg/L in 2007, and it began to decrease thereafter to as low as 1.88 mg/L in 2014. The zooplankton biomass remained stable for the next 5 years. During 2007–2019, the zooplankton biomass declined by 79%.

**Figure 6:**
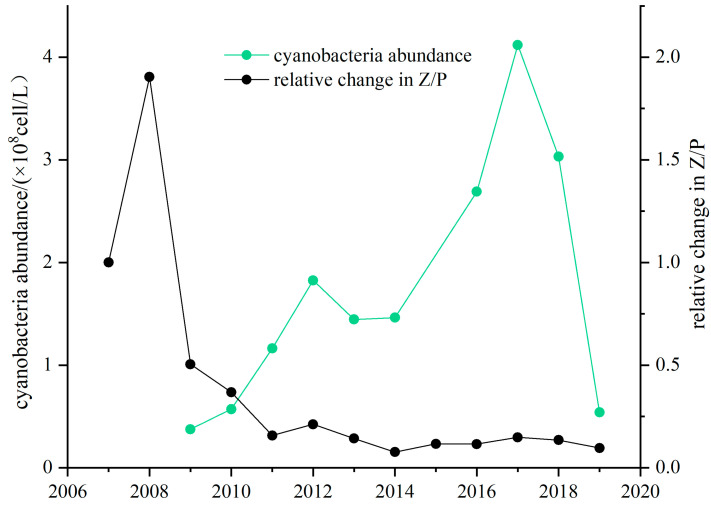
Cyanobacteria abundance and relative change in the Z/P ratio in Lake Taihu.

The Z/P ratio of Lake Taihu decreased dramatically from 2007 to 2019 (Figure 6), and the relationship between the plankton seriously deteriorated. The Z/P ratio of Lake Taihu in 2007 was set as the reference state. The Z/P ratio minimally increased in 2007–2008, with the highest value of 1.90 in 2008. After that, it continuously and significantly decreased in 2008–2011, with the lowest Z/P ratio of 0.16 in 2011. From 2011 to 2019, the Z/P ratio continued to fluctuate and slightly decrease, with a minimum value of 0.08 in 2014. The predatory relationship between the plankton was seriously degraded during these years, and the Z/P ratio was reduced to nearly 1/10 of the initial state.

Cyanobacterial abundance gradually increased after 2009, from only 0.38 × 10^8^ cell/L in 2009 to as high as 4.12 × 10^8^ cell/L in 2017. Cyanobacteria continued to increase from 2009–2012, reaching 1.83 × 10^8^ cell/L by 2012. There was little change from 2012–2014, after which the cyanobacteria began to increase again until it peaked in 2017.

**Figure 7:**
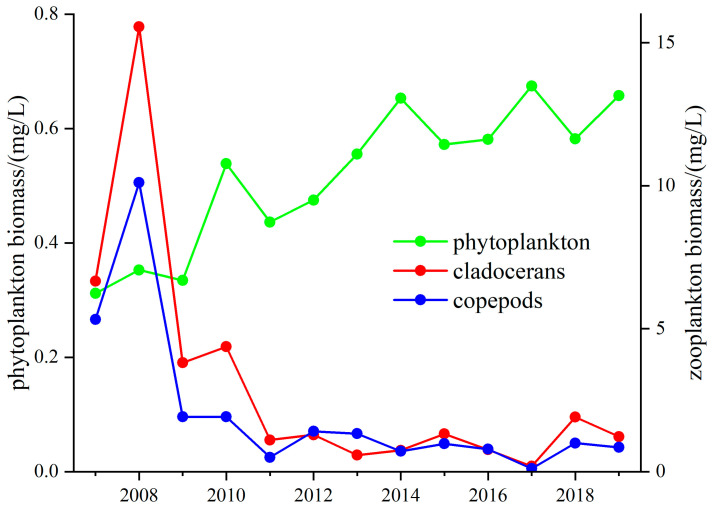
Long-term changes in the cladocerans, copepod, and phytoplankton biomass in Lake Taihu.

The relationship between the crustacean zooplankton and phytoplankton biomass in Lake Taihu changed in 2012. Before 2012, the crustacean zooplankton and phytoplankton biomass were significantly positively correlated (r = 0.49, *p* < 0.05), but after 2012, they were significantly negatively correlated (r = −0.71, *p* < 0.05). The biomass of copepods decreased by 26%, and that of cladocerans decreased by 80.6% during 2008–2012; this change was similar to that for phytoplankton. After 2012, the changes between the crustacean zooplankton and phytoplankton were different, with growth in the phytoplankton biomass accompanied by a fluctuating decrease in the crustacean zooplankton biomass (Figure 7). In 2017, algal blooms were severe, and the phytoplankton biomass was elevated by 29%, while the copepod and cladoceran biomass declined by 86% and 75%.

#### 3.3.2. Lake Donghu

The phytoplankton biomass in Lake Donghu underwent several fluctuations during 2007–2019, while the zooplankton biomass slightly increased. The phytoplankton biomass was 0.58 mg/L in 2007, and then it declined to 0.24 mg/L in 2009. Then, it rebounded rapidly in 2010 and remained stable for the next 5 years. The phytoplankton biomass fluctuated sharply from 2015–2017 but returned to steady-state again after 2018. The zooplankton biomass was 0.95 mg/L in 2007, and it fluctuated consistently over the next 6 years before recovering to 1.31 mg/L in 2012. In the following 7 years, the zooplankton biomass increased slightly, from 1.31 mg/L in 2012 to 2.29 mg/L in 2019.

**Figure 8:**
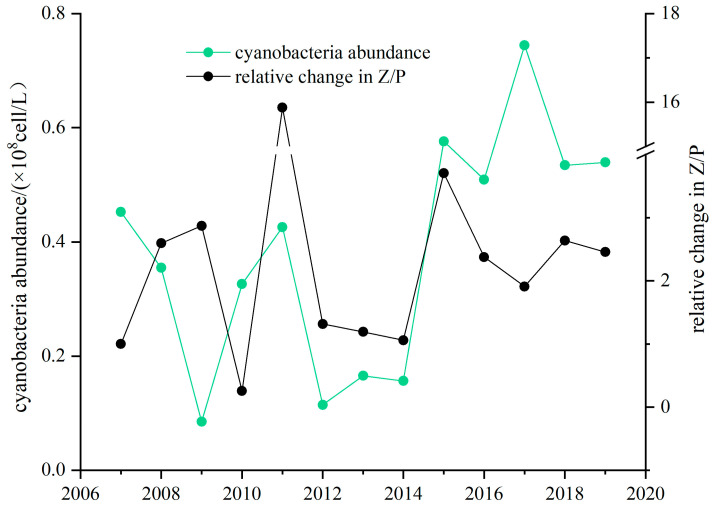
Cyanobacteria abundance and relative change in the Z/P ratio in Lake Donghu.

The increase in the Z/P ratio in Lake Donghu from 2007 to 2019 indicated stable coupling between the plankton. The Z/P ratio of Lake Donghu in 2007 was set as the reference state. The Z/P ratio fluctuated greatly from 2007 to 2012. The Z/P ratio was 1.32 in 2012, and it remained stable in the following 2 years. After 2014, the Z/P ratio increased, reaching 2.46 in 2019 (Figure 8). Overall, coupling between the plankton remained stable from 2007–2019.

Cyanobacterial abundance in Donghu Lake varied little during 2007–2019. The year 2009 had the lowest cyanobacterial abundance at 0.09 × 10^8^ cell/L, while 2017 had the highest value at 0.74 × 10^8^ cell/L. Cyanobacterial abundance increased slightly over the 12-year period.

**Figure 9:**
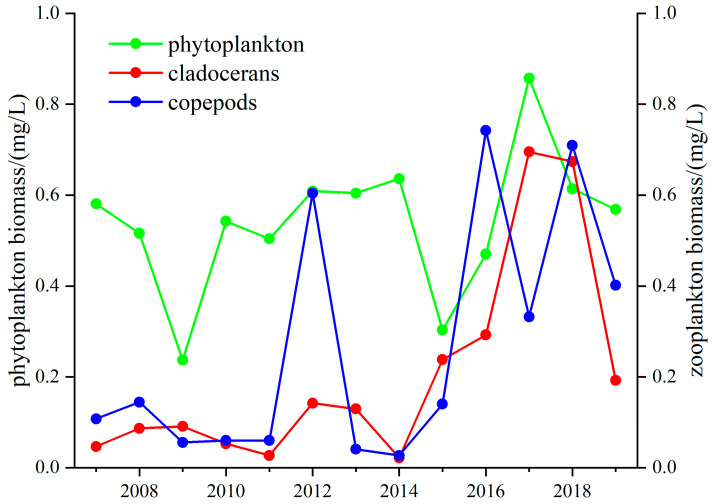
Long-term change in the cladoceran, copepod, and phytoplankton biomass in Lake Donghu.

The crustacean zooplankton biomass in Lake Donghu increased during 2007–2019, and this change was similar to that for phytoplankton. During 2007–2011, the crustacean zooplankton biomass declined overall, with the biomass of the cladocerans and copepods decreasing by 38.6% and 39.8%, while the phytoplankton biomass declined by only 13.8% (Figure 9). The crustacean zooplankton biomass fluctuated from 2011 to 2014 but remained stable overall, and that of the phytoplankton changed in the same way. The zooplankton biomass increased sharply in 2014–2019, with those of both the cladocerans and copepods increasing about 10-fold, while the phytoplankton biomass fluctuated minimally during the same period.

## 4. Discussion

### 4.1. Drivers of Plankton Coupling Relationships in Lakes around the Yangtze River

**Figure 10:**
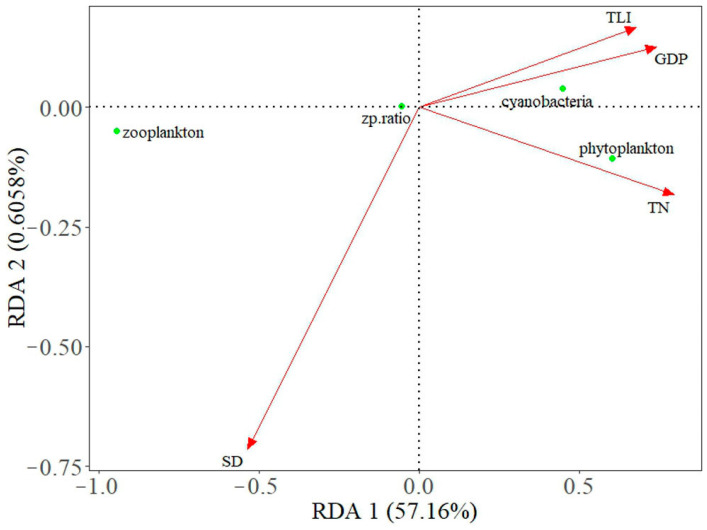
Redundancy analysis of plankton and environmental factors.

Eutrophication due to human activity caused planktonic coupling relationships to deteriorate, phytoplankton densities to dramatically increase, and zooplankton densities to remain almost unchanged, causing a decrease in the Z/P ratio. The phytoplankton densities in the 14 lakes were significantly correlated with the GDP in the lake basins (r = 0.51, *p* < 0.05). Other studies also have shown that the GDP of upstream cities is the key to controlling the trophic levels of lakes [41]. Our data showed that the TLI is significantly correlated with the phytoplankton density in lakes (r = 0.81, *p* < 0.05), which indicates a strong correlation between the eutrophication caused by human and the increase in phytoplankton density in lakes (Figure 10).

Cyanobacterization due to eutrophication weakened the predation pressure of zooplankton on phytoplankton and affected the top–down regulatory function, which led to a decrease in the Z/P ratio [42]. The percentage of cyanobacteria in lakes in the middle and lower reaches of the Yangtze River averaged 42% in the 1990s, but it was as high as 65% in the 2010s (Figure 3). The percentage of cyanobacteria was significantly correlated with the GDP/lake surface area (r = 0.51, *p* < 0.05), which shows that the large amount of nutrients discharged by human activity changed the phytoplankton community structure, so cyanobacteria dominance occurred. Cyanotoxins produced by cyanobacteria limit the growth and reproduction of zooplankton, causing the zooplankton community to change [43]. Due to the increase in cyanobacteria, the density of zooplankton in the lakes in the lower reaches of the Yangtze River remained almost unchanged during the 1990s–2010s (Figure 4). The changes in the proportion of cyanobacteria were significantly negatively correlated with changes in the zooplankton over 20 years (r = −0.79, *p* < 0.05), indicating that there was a strong correlation between the increase in cyanobacteria and the decrease in the zooplankton density (Figure 10). In other words, changes in phytoplankton community structure due to eutrophication are a trigger for the decrease in the Z/P ratio.

The lakes in the middle reaches of the Yangtze River are greatly influenced by the condition of the lake–Yangtze connection. The construction of water conservancy facilities, reclamation, and siltation have altered the hydrological rhythms of many lakes and affected the planktonic coupling relationship [44]. There is high variability in water levels under natural conditions, but water conservancy facilities reduce this variability. There are approximately 50,000 reservoirs in the Yangtze River Basin, especially in the middle and upper reaches where the vertical drop is large. These reservoirs facilitate power generation and flood control, but they also affect the survival of zooplankton. Different zooplankton respond differently to water level fluctuations, and rotifers with short developmental periods are less likely to be affected [45]. The density of rotifers in Lake Changhu and Lake Liangzi increased 6-fold during the 1990s–2010s (Figure 4), which largely contributed to the increase in zooplankton. However, crustacean zooplankton are more sensitive due to their long growth cycle, so changes in hydrological rhythms may threaten these zooplankton and inhibit their ability to control phytoplankton. Water level fluctuations impact the growth of aquatic plants. The change in the lake–Yangtze connection has led to the degradation of aquatic plants, while phytoplankton dominate [46]. The phytoplankton density of all five lakes in the middle reaches of the Yangtze River increased during the 1990s–2010s (Figure 3).

The ecosystems of plateau lakes are more fragile than those of plain lakes, and human activities and biological invasions are the most important causes of changes in plankton relationships. Most of the lakes in the upper reaches of the Yangtze River are plateau lakes, such as Lake Dianchi and Lake Erhai. The RUE of phytoplankton in plateau lakes is much higher than that in plain lakes due to the influence of geography and the climate [47]. In recent years, algal blooms in plateau lakes have been exacerbated due to human discharge, with cyanotoxins and reduced dissolved oxygen concentrations harming zooplankton. Meanwhile, plateaulakes are also more sensitive to climate change than plain lakes are, which further accelerates ecosystem degradation. The phytoplankton density, especially of the cyanobacteria of Lake Dianchi, has been consistently high for 20 years, and the phytoplankton density of Lake Erhai also increased during this period (Figure 3). This is related to the pollution caused by human activity in the watershed [48]. Due to the demands of fisheries, humans have stocked many plateau lakes with exotic fish, especially zooplanktivorous fish [49]. These fish threaten the survival of indigenous fish and prey upon zooplankton, especially crustacean zooplankton. The percentage of crustacean zooplankton in Lake Erhai reached 26% in the 1990s, whereas it plummeted to 3% in the 2010s, while the density of rotifers increased 6-fold (Figure 4). The predation pressure exerted by invasive fish on zooplankton may be an important reason for this phenomenon.

### 4.2. Differences in Plankton Coupling Relationships between Lake Taihu and Lake Donghu

Due to eutrophication and cyanobacterial blooms, a significant decrease in the Z/P ratio occurred in Lake Taihu during 2007–2019. In contrast, fewer cyanobacterial blooms occurred in Lake Donghu, which kept the Z/P ratio stable during the same period.

Nutrient increase was the main reason for the sharp increase in phytoplankton in Lake Taihu during 2007–2019. The slight growth of zooplankton was mainly due to cyanobacterial blooms, which, together, led to a large decrease in the Z/P ratio and the degradation of the plankton coupling relationships. Phytoplankton proliferation was the primary factor leading to the degradation of the plankton coupling relationships. The phytoplankton biomass gradually increased from 2007 [2]. The changes in hydrometeorological conditions further increased the intensity and number of algal blooms. Global warming has increased the water temperatures and decreased the wind speeds in Lake Taihu, which are favorable for the proliferation of cyanobacteria [50]. The rapid development of the Lake Taihu Basin has resulted in high nutrient inputs into the lake, and our data show that the Z/P ratio decreased with increasing total phosphorus concentration, which is similar to the findings of other researchers [51]. Our data also showed that the Z/P ratio was significantly and positively correlated with the TLI in the years prior to 2012 (r = 0.81, *p* < 0.05), which indicated that the phytoplankton were controlled by the zooplankton until 2012.

The analysis showed that the zooplankton biomass in Lake Taihu was significantly negatively correlated with cyanobacterial occupancy (r = −0.59, *p* < 0.05), which indicated a strong correlation between increased cyanobacterial occupancy and zooplankton reduction. Cyanobacteria and the Z/P ratio were significantly negatively correlated from 2009–2014 (r = −0.71, *p* < 0.05), indicating a strong correlation between an increased cyanobacteria population and a decreased Z/P ratio. The biomass of crustacean zooplankton declined significantly from 2008 and was consistently low after 2011, resulting in less predation pressure on the phytoplankton (Figure 7). Cyanobacteria release cyanotoxins which not only harm zooplankton but also miniaturize them. This change results in the reduced predation pressure of zooplankton on phytoplankton. Moreover, global warming cause zooplankton to decrease in size at maturity, further exacerbating the trend toward smaller zooplankton [52]. In Lake Taihu, the structure of the fish community does not favor zooplankton survival, with dominant species such as *Neosalanx taihuensis* putting predatory pressure on crustacean zooplankton. Other researchers have found that removing piscivorous small fish can significantly increase the Z/P ratio, simultaneously enlarging the size of cladoceran zooplankton and enhancing the top–down control of zooplankton on phytoplankton [53]. The absence of mixed fish and shrimp also adversely affects the growth of zooplankton [8,54].

The control of phytoplankton by silver and bighead carp is the primary reason for the stable Z/P ratio in Lake Donghu over the last two decades. Since the 1990s, despite consistent eutrophication, Lake Donghu has rarely experienced cyanobacterial blooms. Extensive research has demonstrated that the predation pressure exerted by fish such as silver carp and bighead carp has reduced the risk of cyanobacterial blooms in lakes [55,56]. Analysis has revealed no correlation between the Z/P ratio, TLI, and the biomass of phytoplankton, indicating that the TLI is not the main cause of the changes in the Z/P ratio. Figure 8 also shows that the biomass of phytoplankton in recent years has remained stable, and there was no significant correlation between the cyanobacteria and the Z/P ratio (*p* > 0.05). Predation by fish not only reduces the likelihood of cyanobacterial blooms but also provides a favorable habitat for zooplankton, enhancing their predation on phytoplankton. Figure 9 indicates that the biomass of crustacean zooplankton increased from 2007, especially after 2014. This phenomenon suggests that zooplankton in Lake Donghu continue to exert significant predation pressure on phytoplankton.

### 4.3. Suggestions for Lake Management

Increased phytoplankton and cyanobacterization were important factors contributing to the degradation of the planktonic coupling relationship, so the cyanobacteria population needs to be reduced by inhibiting exogenous and endogenous pollution. Restoring fish community structures, removing invasive species, restoring aquatic plants, and improving river–lake relationships favor zooplankton restoration. This combination of actions will facilitate the restoration of plankton coupling relationships.

In recent years, China has emphasized the ecological and environmental management of the Yangtze River. Many lakes in the middle and lower reaches of the Yangtze River are under the effective control of exogenous pollution, and nutrient concentrations are declining [57]. A number of small water conservancy facilities have been removed from the middle and upper reaches of the Yangtze River [58]. The managers of some plateau lakes continue to harvest exotic fish. All of these actions favor the restoration of planktonic coupling relationships; we believe that plankton coupling relationship in lakes around the Yangtze River will become better, but endogenous pollution control and the removal of exotic fish still need to be improved.

## 5. Conclusions

During the 1990s–2010s, among the 14 lakes analyzed, 11 exhibited a lower growth rate of zooplankton densities compared to those of phytoplankton, with a substantial decline in the Z/P ratio, averaging 92%. Over these two decades, 79% of the lakes saw significant increases in the phytoplankton density, with an average increase of 3,156-fold, while only 57% of the lakes experienced increases in the zooplankton density, with an average increase of only 2-fold. Overall, the zooplankton population grew less rapidly than that of the phytoplankton. In many lakes, the predation pressure of zooplankton on phytoplankton decreased, leading to the decoupling of the lake food webs. Eutrophication led to an increase in phytoplankton. Cyanobacterization and changes in the fish community structure restricted zooplankton growth. The combination of these two causes resulted in a drop in the Z/P ratio and a decrease in the zooplankton RUE. Additionally, climate change has had a significant impact on the Z/P ratio in most lakes. Cyanobacterization causes the Z/P ratio to decrease, so it is necessary to pay attention to nutrient management. In addition, the restoration of predatory fish and the proper management of filter-feeding fish can help restore plankton communities.

## Figures and Tables

**Table 1:** Lakes and references involved in this study.

Lake	Lake Area (km^2^)	Lake Basin GDP in 2010s (billion CNY)	Data Sources
Lake Yangcheng	120	634	[17,18]
Lake Taihu	2340	224	CNERN
Lake Shijiu	208	2080	[19,20]
Lake Hongze	2069	430	[21,22]
Lake Luoma	296	785	[23,24]
Lake Chaohu	780	441	[25,26,27]
Lake Caizi	226	94	[28,29]
Lake Poyang	3150	645	[30,31,32]
Lake Donghu	32	546	CNERN
Lake Liangzi	370	179	[33,34,35]
Lake Changhu	131	308	[36]
Lake Dongting	2579	365	proprietary dataset
Lake Dianchi	330	217	[37,38,39]
Lake Erhai	250	91	proprietary dataset

**Table 2:** $r_{ij}^{2}$ parameters.

	*chla*	*TP*	*TN*	*SD*	*COD_Mn_*
$r_{ij}^{2}$	1	0.7056	0.6724	0.6889	0.6889

## Data Availability

Data are derived from public domain resources.
